# Chronic thoracic pain after cardiac surgery: role of inflammation and biomechanical sternal stability

**DOI:** 10.3389/fpain.2023.1180969

**Published:** 2023-08-11

**Authors:** Jeko M. Madjarov, Michael G. Katz, Yoav Hadas, Sofia Jisoo Kim, Lina Freage-Kahn, Svetozar Madzharov, Adam Vincek, Sophia J. Madjarova, Piers Seidman, Nataly Shtraizent, Steven A. Robicsek, Efrat Eliyahu

**Affiliations:** ^1^ Department of Cardiovascular Surgery, Wake Forest School of Medicine, Winston-Salem, NC, United States; ^2^ Department of Cardiovascular Surgery, Atrium Health Sanger Heart and Vascular Institute, Charlotte, NC, United States; ^3^ Department of Genetics and Genomic Sciences, Icahn School of Medicine at Mount Sinai, New York, NY, United States; ^4^ Department of Cardiovascular Surgery, Icahn School of Medicine at Mount Sinai, New York, NY, United States; ^5^ Department of Biology and Environmental Studies, New York University, New York, NY, United States; ^6^ Frezent Biological Solutions, New York, NY, United States; ^7^ Department of Biology, Columbia University, New York, NY, United States; ^8^ Baruch College, City University of New York, New York, NY, United States; ^9^ Senex, New York, NY, United States; ^10^ Department of Anesthesiology, College of Medicine, University of Florida, Gainesville, FL, United States; ^11^ Icahn School of Medicine at Mount Sinai, Icahn Genomics Institute, New York, NY, United States

**Keywords:** cardiac surgery, chronic chest pain, mediastinitis, cytokines, chest reconstruction, sternal healing

## Abstract

**Introduction:**

The pathogenesis of chronic chest pain after cardiac surgery has not been determinate. If left untreated, postoperative sternal pain reduces the quality of life and patient satisfaction with cardiac surgery. The purpose of the study was to examine the effect of chest inflammation on postoperative pain, risk factors for chronic pain after cardiac surgery and to explore how chest reconstruction was associated with the intensity of pain.

**Methods:**

The authors performed a study of acute and chronic thoracic pain after cardiac surgery in patients with and without sternal infection and compared different techniques for chest reconstruction. 42 high-risk patients for the development of mediastinitis were included. Patients with mediastinitis received chest reconstruction (group 1). Their demographics and risk factors were matched with no-infection patients with chest reconstruction (group 2) and subjects who underwent conventional sternal closure (group 3). Chronic pain was assessed by the numeric rating scale after surgery.

**Results:**

The assessment of the incidence and intensity of chest pain at 3 months post-surgery demonstrated that 14 out of 42 patients across all groups still experienced chronic pain. Specifically, in group 1 with sternal infection five patients had mild pain, while one patient experienced mild pain in group 2, and eight patients in group 3. Also, follow-up results indicated that the highest pain score was in group 3. While baseline levels of cytokines were increased among patients with sternal infection, at discharge only the level of interleukin 6 remained high compared to no infection groups. Compared to conventional closure, after chest reconstruction, we found better healing scores at 3-month follow-up and a higher percentage of patients with the complete sternal union.

**Conclusions:**

Overall, 14 out of 42 patients have chronic pain after cardiac surgery. The intensity of the pain in mediastinitis patients significantly decreased at 3 months follow-up after chest reconstruction. Thus, post-surgery mediastinitis is not a determining factor for development the chronic chest pain. There is no correlation between cytokines levels and pain score except interleukin 6 which remains elevated for a long time after treatment. Correlation between sternal healing score and chronic chest pain was demonstrated.

## 1. Introduction

According to recent national study, the total cardiac surgery procedures volume in USA is expected to continue growing, reaching 1.3 million annual procedures in 2026 (1, 2). Although minimally invasive and robotic cardiac surgery is an attractive choice for patients, more than 95% of surgeons preferred to use the median sternotomy incision because it provides optimal exposure and access to the entire heart. However, one of the consequences after sternotomy is the development of chronic thoracic pain (CTP) affecting 17%–56% of patients (3–5). CTP has been defined by the International Association for the study of pain as a non-anginal persistent postoperative pain lasting more than 2–3 months post-surgery without other causes of pain such chronic condition preceding the surgery. It may present as numbness, allodynia, palpation tenderness, or constant pain (6). Factors that aggravate CTR include pressure at the site, clothes rubbing against the scar, movement, deep breathing, coughing, weather/temperature change, and stress (7). The incidence of chronic post-sternotomy pain is underestimated (8). Given the considerable anxiety and fear associated with cardiac surgery, patients do not wish to complain about pain, which they think as “trivial” or “expected” (9). Without proper diagnosis and treatment, chronic sternal pain decreases the quality of life affecting sleep, mood, and activity level. The exact etiology of CTP is unknown. There are numerous hypotheses have been considered, including entrapment neuropathy caused by sutures or scar tissue at the site of sternotomy, and intercostal neuralgia resulting from damage to the intercostal nerves during the dissection of the internal mammary artery (10). Also, it has been suggested that CTP can be a result of rib fractures related to the surgical procedure, and costo-sternal syndrome. Incomplete sternal healing and wound inflammation/infection can be associated with CTP as well (11).

Another important current problem associated with thoracic pain after surgery is a new persistent opioid use. Unfortunately, patients undergoing open thoracic/cardiac surgery had the highest rate of prolonged opioid use compared with other surgical cohorts (12). Others and we believe that improving the usual surgical techniques of the chest closing will minimize the risk of postoperative neuropathic pain (13, 14).

To the best of our knowledge, detailed pain assessment in acute chest inflammation and the healing process of deep sternal infection was not reported before. Deep sternal wound infection with mediastinitis is one of the most devastating complications after cardiac surgery (15). Mediastinitis typically occurs approximately 3–10 days after surgery and is usually led to sternal wound dehiscence and frank purulent drainage. This complication remains a significant source of mortality (up to 25%), treatment usually includes repeated hospitalization with secondary surgical procedure like as bone cleaning and debridement, vacuum assisted therapy, transposition of muscle flap or greater omentum, and long-time reception of different classes of antibiotics (16). Deep sternal infection is associated with surgical trauma to chest tissue and mediastinal organs, as well as the presence of an open surgical wound. Chest tissue inflammation leads to neurogenic inflammation at the site of the surgical trauma. It changed the sensitivity of primary afferent nerve terminals (“peripheral sensitization”) and the central nervous system (“central sensitization”) (17).

The primary aim of the study was to examine the risk factors for chronic thoracic pain after cardiac surgery in patients with deep sternal infection and to assess the link between inflammatory biomarkers, and chest pain. A secondary aim was to validate if the newly suggested chest reconstruction technique can improve sternal healing and reduce post-cardiac surgery pain.

## 2. Methods

### 2.1. Study population and sample size

In this study, we included 42 patients who were at an increased risk of developing deep sternal infection (mediastinitis) after undergoing cardiac surgical procedures. Of these patients, 14 developed deep sternal infection (mediastinitis with sternal dehiscence), as defined by the acceptable criteria described below in the Results section 3.2. Clinical description of patients with deep sternal infection. All these patients underwent conventional wire closure during their first cardiac surgery procedure, which was defined as passing six or more wires transsternally, with transsternal sutures passed approximately 1 cm on each side to reinforce the lateral table of the sternum. The suture wires were then crossed, pulled, and twisted. After developing mediastinitis, these 14 patients received longitudinal chest wall reconstruction (LCWR) as a treatment (group 1). We matched their demographics and a risk factor with another 14 high-risk patients for the development of deep sternal infection (group 2) who received primary LCWR with no signs of post-surgery infection, as well as with another 14 high-risk subjects who underwent conventional wire closure (CWC) with no signs of post-surgery infection (group 3).

Patients were followed up clinically and the pain was assessed both during their hospital stay and in the outpatient follow-up setting. The patient's screening consisted of a physical examination including an assessment of wound healing, CT imaging, a personal interview about pain level, and molecular analysis of cytokines proinflammatory factors and C-reactive protein (CRP). All patients received a similar anesthesia protocol that is routinely used in our department.

The postoperative analgesia regimen included HYDROmorphone (DILAUDID) injection of 0.5 mg intravenous and an Oxycodone (Roxicodone) tablet of 5–10 mg. Acetaminophen (paracetamol) and acetaminophen–codeine tablets (500 mg acetaminophen–30 mg codeine) were used as adjunctive analgesics. Some patients received non-steroidal anti-inflammatory drugs (NSAD). However, many doctors avoid prescriptions for NSAD because of concerns about bleeding and renal dysfunction.

### 2.2. Study design

The Institutional Review Board at the Atrium Health Sanger Heart and Vascular Institute and Wake Forest Medical School approved and reviewed the study. All patients were informed and gave written consent. The current study is case-control. Group 1 and 3 were retrospective studies because we collected the information about patient's past. In group 2 patients are followed over time and data about them is collected as their characteristics in prospective manner.

#### 2.2.1. Matching criteria

The groups matching was based on the number of patients, the technique of chest closure after surgery, demographic characteristics, and comorbidities such as diabetes, hypertension, chronic obstructive pulmonary disease (COPD), and choice of cardiac procedure such as coronary artery bypass surgery (CABG), valvular or aortic procedure.

### 2.3. Data collection

Demographics and clinical characteristics were collected from the hospital's electronic health record. All patients participated in individual interviews with clinicians focused on pain intensity and pain management. Patient interviews were scheduled on day one, the day of discharge, one month, and three months after surgery.

### 2.4. Diagnosis of deep sternal infection (mediastinitis)

Diagnosis of deep sternal infection was based on the following findings: wound tenderness, purulent drainage, cellulitis, fever with or without leukocytosis, and sternal instability. All patients with deep sternal wound infections were classified according to the accepted criteria (18) including (a) isolation of the microorganism in the culture of mediastinal bone tissue or of liquid obtained through fine-needle aspiration or during the surgery, (b) evidence seen during surgery or through histopathologic evaluation, (c) presence of one of the following clinical findings: fever, chest pain, sternal instability, draining of purulent secretion of the mediastinal area, and widening of the mediastinum detected through radiologic evaluation.

### 2.5. The technique of chest wall reconstruction by longitudinal bilateral rigid plating

Surgical details of chest wall reconstruction have been previously described (19). Briefly, soft tissue overlying the sternum was mobilized to expose the entire length of the bone. Preserving the periosteum, the attachments of the pectoralis major muscles were partially detached. Lateral exposure of the sternum facilitates the proper application of bone reduction clamps and calipers. This allows for accurate measurement of bone depth. CT scan and lateral chest x-ray also measured the thickness of the sternum. We used a power screwdriver, which locks the screws into the plate. The periosteum was left intact. The goal is to support the sternum in its entire length. Two plates were used, one on each side of the fracture. Once the plates have been fully secured, we tighten all the screws with a screwdriver.

In cases of deep sternal wound infection (DSWI) accompanied by sternal fragmentation and osteomyelitis, we sometimes opt for sternectomy and closure of soft tissues using various flaps. Alternatively, in select cases of DSWI with fragmentation, we perform minimal sternal debridement. If the tissue appears healthy, we proceed with longitudinal rigid fixation. Following multiple washouts, wound vac therapy, and attainment of negative cultures, we proceed with definitive closure. Our approach has demonstrated favorable long-term outcomes in carefully selected patients. Although not routine, considering PET scans in specific cases may be worth considering. One notable advantage of our technique is the expedited re-entry process. Our plates are securely affixed along the lateral edge of the sternum, approximating the sternal halves using regular cerclage wires. This configuration protects the sternum from wire-induced damage. In the event of re-entry, the wires can be readily cut, simplifying the procedure. Another advantage is that the plates remain in place during re-entry, allowing for closure using new wires (20).

### 2.6. Possible pre-, intra-, and postoperative risk factors for the chronic thoracic pain

Possible pre- and intraoperative risk factors were analyzed as independent predictors of CTP. These factors included insulin-dependent diabetes mellitus, obesity based on body mass index, chronic obstructive pulmonary disease (COPD), previous sternotomy, arterial hypertension, peripheral vascular disease, renal failure, recent myocardial infarction up to 1 month before the surgery, unstable angina, and immunosuppressed state. Intra- and postoperative risk factors included duration of the surgery, cardiopulmonary bypass, use of internal mammary arteries, and length of intensive care unit (ICU) stay.

### 2.7. Thoracic pain assessment and management

The pain was assessed with a numeric rating scale (NRS) at rest, and on movement using an 11-point scale with 0 labeled as “no pain” and 10 as “worst pain imaginable”. The pain was classified as mild with a score of 1–3, moderate with scores of 4–6, and severe with scores of 7–10. After the operation, the actual pain levels were assessed at four time points. All patients were instructed to use the pain-scoring tool correctly and the physician-investigator carefully checks to differentiate chronic chest pain after surgery from angina or another cardiac pain. The pain was relieved by medication including opioids to achieve patients' satisfaction.

### 2.8. Cytokines level and C-reactive protein measurement

Six cytokines, including interleukin (IL) 1,6,17, tumor necrosis factor (TNF α), interferon (IFN γ), RANTES, and C-reactive protein (CRP) were analyzed for a two-time point at admission and discharge for each study group. Quantified human ELISA kits were purchased from R&D systems (Minneapolis, MN). Analyses were performed according to the manufacturer's protocol for each ELISA kit, assayed in triplicate, and read on a Molecular Devices microplate reader at 450 nm (Menlo Park, CA). Standard curves and individual good concentrations were determined using the Softmax 2.34 software. To keep experimental values within the linear region of the standard curves, samples were diluted from 1:5 to 1:100 as necessary for stimulated culture samples.

### 2.9. Blood collection

Blood samples for cytokine and CRP analysis were collected through venipuncture at the admission and discharge. The collected blood samples were transferred to Eppendorf tubes and placed on ice during transport to the laboratory. Following centrifugation of the samples at 1,500 rpm for 10 min at 4°C, the serum was carefully collected from each sample and stored at −80°C until the analysis.

### 2.10. Assessment of sternal bone healing

All CT scan examinations were performed at 3-month postoperative period using a multi-detector computed tomography scanner. No specific patient preparation was required apart from quiet breathing. Patients were scanned in a supine position. The scan started below the diaphragm and ended at the supraclavicular regions. Cardiothoracic surgeon and radiologist conducted an analysis of CT scans from patients with chest reconstruction or wires. Axial slices were analyzed using a point quantitative scale (0 = no sign of healing, 1 = minimal healing, 2 = mild healing, 3 = moderate healing, 4 = partial synthesis, 5 = complete synthesis) as previously described (14, 20, 21). The sternal union was defined as a mean score of 3 or greater, which indicates sternal stability and the healing process.

### 2.11. Statistical analysis

Statistical analyses were performed with SPSS 11.0 software (SPSS, Inc, Chicago, IL). Univariate analyses were performed for all relevant categorical variables by using contingency tables (*χ*^2^) or Fisher exact tests for variables with small numbers) and *t*-tests for continuous variables. Multivariate analyses were performed by using a stepwise logistic regression, in which all variables with a *p*-value of less than 0.10 in the univariate analyses or that were thought to be related to chest pain from the literature were included in the initial full models. A *p*-value of less than 0.05 was considered significant.

#### 2.11.1. Hazard ratio

We are performing a hazard ratio analysis on the data to determine whether there is a higher chance of experiencing high pain in different groups at several time points. For the high pain level category, pain score of 7–10, the hazard ratio was calculated by finding the quotient between the group with LCWR and CWC high pain patients divided by the total number of patients in the study for each group. This ratio was calculated for every time period: 1 day, discharge, 1 month, and 3 months. This was done for all groups at rest and at movement.

## 3. Results

### 3.1. Demographic, clinical characteristics, preoperative and intraoperative risk factors

Among 42 patients, there were 27 males and 15 females; the mean age of patients was 67 ± 2.45 years. There was no significant difference in demographic characteristics between the subjects in all groups. Forty-four percent of all patients were with body mass index (BMI) of over 30 kg/m^2^, and 14.2% were with BMI of over 40 kg/m^2^. Preoperative and intraoperative characteristics associated with chronic chest pain are outlined in Table 1. The difference in operation time was not significant changes in all groups (*p* > 0.05). All patients were presented with primary cardiac surgery. For patients in group 1 chest reconstruction was a secondary procedure after cardiac surgery. Twenty-six patients (61.9%) had undergone coronary artery bypass grafting, 6 (14.2%) had valvular procedures and 8 (19%) had aortic surgery. There were no significant differences between groups in terms of possible preoperative risk factors such as diabetes mellitus, chronic obstructive pulmonary disease, hypertension, tobacco usage, BMI, and immunosuppressive state. Among psychosocial factors patients with mediastinitis had a higher percent of anxiety and pain history. Clinical characteristics and postoperative variables of the patients are presented in Table 2. We did not see postoperative complications in these cohorts of patients. There was no in-hospital and 3-month post-surgery mortality in all groups. Patients in group 1 had prolonged intensive care unit (ICU) stays however it was not significantly different from other groups (*p* > 0.05). The mean hospital stays for groups 1, 2, and 3 were respectively 11.5 ± 5.42 days, 6.42 ± 1.39 days, and 6.5 ± 0.94 days (*p* = 0.0022). The data demonstrate the significant difference in the dose of opioids received by patients in ICU in the mediastinitis group compared with no infection groups (*p* = 0.0004). However, this difference disappeared when the patients moved to the surgical ward after the procedure (*p* > 0.05). At 3 months post-surgery four patients (3 in the third group and 1 in the first group) confirmed that they were still taking Oxycodone 5 mg once a day. Nine patients in group 1 received Oxycodone before admission to the hospital.

**Table 1:** Preoperative and intra-operative risk factors for chest pain by univariate analysis.

Groups	Mediastinitis LCWR group 1	No infection LCWR group 2	No infection CWC group 3	*p* Value
Demographics				
Age, years	67.5 ± 5.99	67.1 ± 6.03	69.7 ± 6.58	0.37
Sex distribution, male	9 (64.2%)	9 (64.2%)	9 (64.2%)	>0.99
Hypertension	5 (35.7%)	4 (28.5%)	5 (35.7%)	>0.99
Diabetes	21.4%	28.5%	21.4%	>0.99
COPD	2 (14.2%)	1 (7.14%)	2 (14.2%)	>0.99
Current tobacco use	3 (21.4%)	4 (28.5%)	3 (21.4%)	0.67
Immunosuppressive state	3 (21.4%)	2 (14.2%)	2 (14.2%)	0.63
Psychosocial factors				
Anxiety	8 (57.1%)	4 (28.5%)	2 (14.2%)	0.083
Depression signs	3 (21.4%)	1 (7.14%)	1 (7.14%)	0.61
Pain history	11 (78.5%)	2 (14.2%)	2 (14.2%)	0.057
Body mass index				
30–35, kg/m^2^	6 (42.8%)	5 (35.7%)	7 (53.5%)	0.71
35–40, kg/m^2^	5 (35.7%)	7 (53.5%)	6 (42.8%)	0.71
>40, kg/m^2^	3 (21.4%)	2 (14.2%)	1 (7.14%)	0.29
Intraoperative variables				
Surgery, min	163.1 ± 14.9	166.6 ± 22.1	155.3 ± 10.7	0.12
CABG	8 (57.1%)	8 (57.1%)	10 (71.4%)	0.69
Valve procedures	2 (14.2%)	2 (14.2%)	2 (14.2%)	>0.99
Aortic procedures	4 (28.5%)	4 (28.5%)	2 (14.2%)	0.37

CABG, coronary artery bypass grafting; COPD, chronic obstructive pulmonary disease; Values presented as *N* (%), Mean ± SD, *p*-values: unpaired *t*-test.

**Table 2:** Postoperative variables for chest pain by univariate analysis.

Groups	Mediastinitis LCWR group 1	No infection LCWR group 2	No infection CWC group 3	*p* Value
ICU, length of stay (h)	38.6 ± 27.7	32.4 ± 13.3	25.4 ± 4.6	0.092
Hospital length of stay (days)	11.5 ± 5.41	6.42 ± 1.39	6.5 ± 0.94	0.002**
Amount of opioids in ICU (mme)	46.4 ± 11.9	30.6 ± 6.51	31.7 ± 6.42	0.0004***
Amount of opioids in surgical ward (mme)	21.7 ± 9.01	15.1 ± 3.54	22.2 ± 4.88	0.87

Mean ± SD, *p*-values: unpaired *t*-test. ICU, intensive care unit; mme, morphine milligram equivalents.

** *p*- value <0.01.

*** *p*- value <0.001.

### 3.2. Clinical description of patients with deep sternal infection

**Figure 1:**
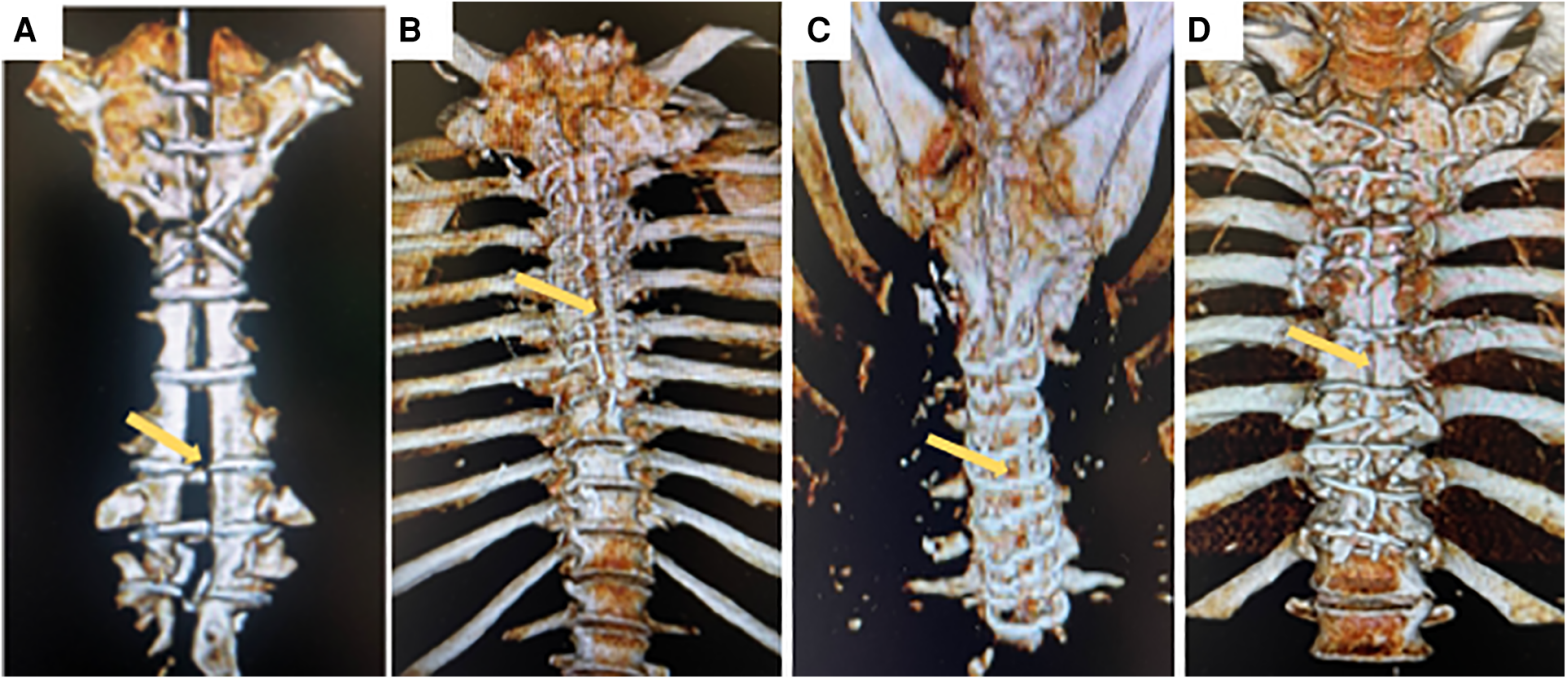
Representative three-dimensional computed tomography scans for patient with deep sternal infection with sternal dehiscence and mediastinitis (yellow arrow) (**A**) and same patient after chest wall reconstruction with complete bone healing (yellow arrow) (**B**). Patient with complete sternal healing after chest reconstruction (**C**), Patient with incomplete sternal healing after conventional sternal closure (**D**).

All 14 patients with deep sternal infection (mediastinitis) were accepted and treated in our institution. The time between the primary surgery date and the onset of inflammatory symptoms ranged from 3 to 11 days. Surgical wound cultures were positive in all cases. Deep sternal infection was caused by a marked inflammatory response characterized by clinical symptoms like as fever, chest pain, sternal instability, draining of purulent secretion, widening of the mediastinum on chest x-ray, isolation of the microorganism in the culture of mediastinal tissue, or blood, and a marked rise in circulating cytokines and C reactive protein in serum. After chest reconstruction surgery clinical symptoms gradually declined, and on discharge day all patients fully recovered (Figures 1A-D).

### 3.3. Assessment of pain severity after cardiac surgery

**Figure 2:**
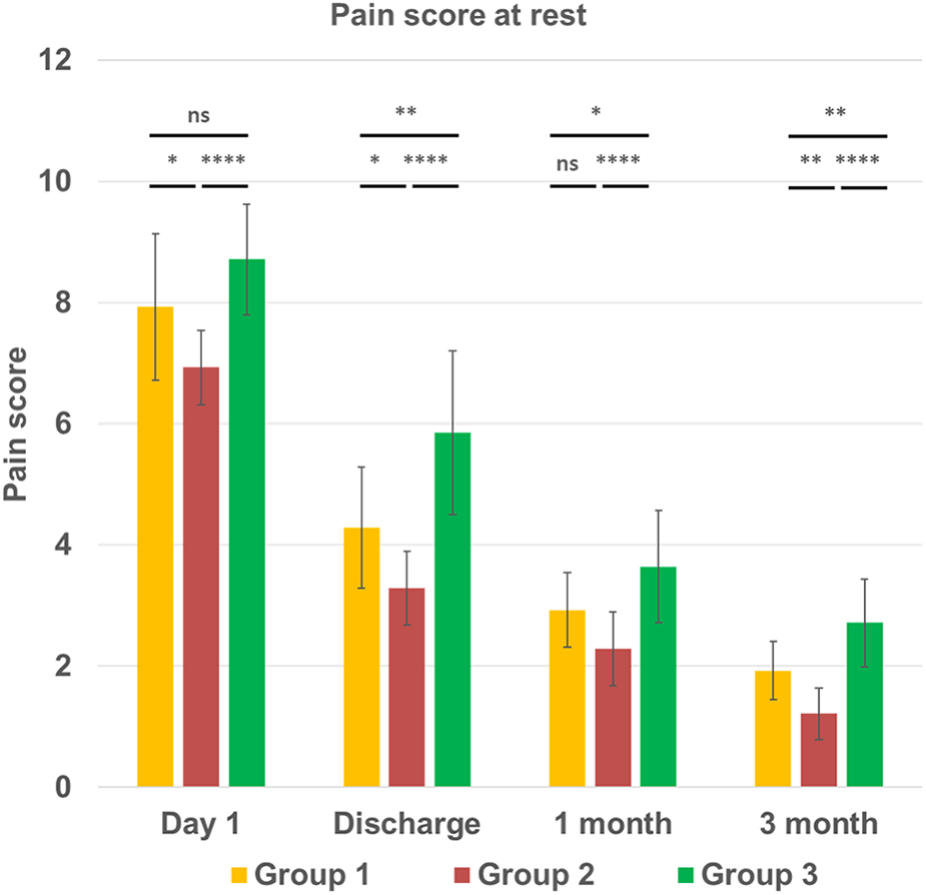
Intensity of the chest pain at rest condition (*vertical line*) at different time points (*horizontal line*) in three groups. All continuous data were checked for normality and are presented as mean ± SD. NS, not significant. *p* > 0.05; *p ≤ 0.05; **p ≤ 0.01; ***p ≤ 0.001; ****p ≤ 0.0001. Group 1 LCWR, longitudinal chest wall reconstruction with mediastinitis, Group 2 LCWR, longitudinal chest wall reconstruction with no infection, Group 3 CWC, conventional wire closure with no infection.

**Figure 3:**
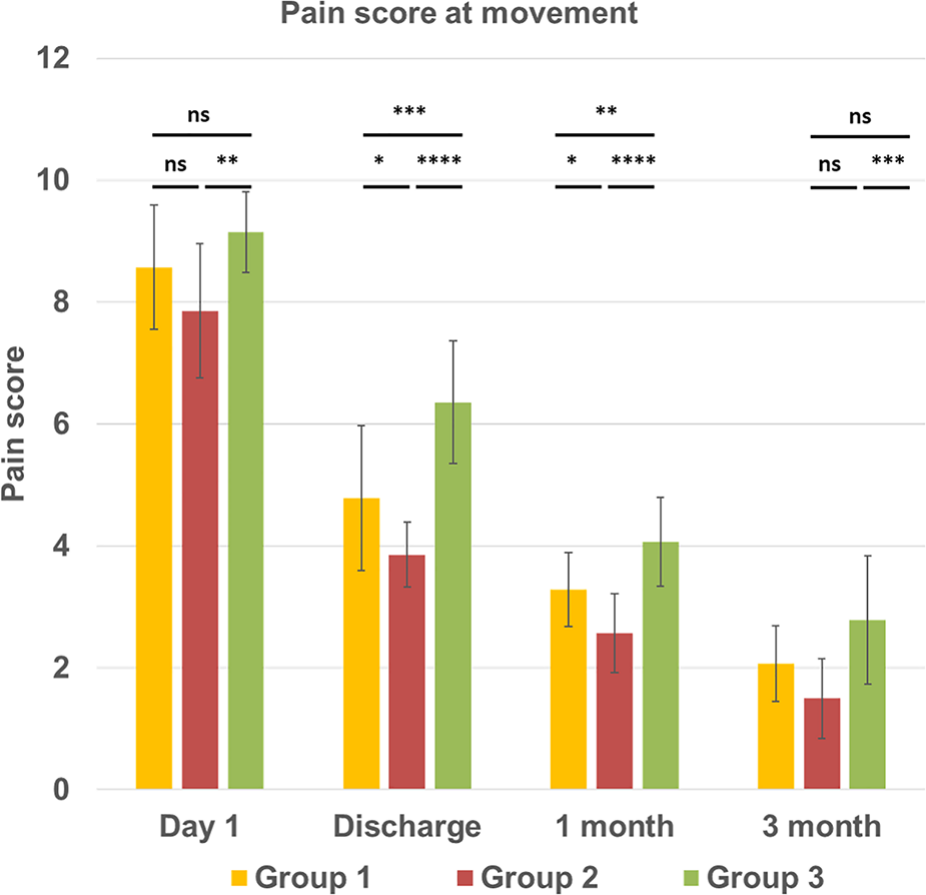
Intensity of the chest pain at movement condition (*vertical line*) at different time points (*horizontal line*) in three groups. All continuous data were checked for normality and are presented as mean ± SD. NS, not significant. *p* > 0.05; *p ≤ 0.05; **p ≤ 0.01; ***p ≤ 0.001; ****p ≤ 0.0001. Group 1 LCWR, longitudinal chest wall reconstruction with mediastinitis, Group 2 LCWR, longitudinal chest wall reconstruction with no infection, Group 3 CWC, conventional wire closure with no infection.

The assessment of the incidence and intensity of chest pain at 3 months post-surgery demonstrated that 14 out of 42 patients across all groups still experienced chronic pain. Specifically, in group 1 with sternal infection five patients had pain, while one patient experienced pain in group 2, and eight patients in group 3. Pain scores for each postoperative period at rest and during movement are displayed in Figures 2, 3. On postoperative day 1, the mean pain score at rest was lowest in group 2, 6.92 ± 0.61, and highest in group 1 (7.92 ± 1.20), and in group 3 (8.71 ± 0.91), with a significantly difference between the groups (*p* < 0.001). The intensity of initial postoperative pain varied significantly between the groups. On the day of discharge, the mean pain score at rest had decreased to 4.78 ± 1.12, 3.92 ± 1.07, and 5.85 ± 1.35 in groups 1, 2, and 3, respectively (group 1 vs. group 2, *p* < 0.05; group 2 vs. group 3, *p* < 0.0001). Interestingly, the results on the day of discharge, one month, and three months follow-up indicated that the pain score for the patients was higher in group 3 with no infection and regular sternal closure (2.71 ± 0.72, *p* < 0.01) but not in the group with the previous inflammatory process with deep sternal infection as expected. The same trend was observed for the pain score during movement. Pain intensity during moving was higher, especially on day 1 and day of discharge. A significant difference was found between groups 2 and 3 at three months follow-up (1.5 ± 0.65 vs. 2.78 ± 1.05, *p* < 0.001). The assessment of chronic pain at the three months follow-up demonstrated that in groups 1 and 2, patients had only mild pain. In group 3, three out of 14 patients had moderate pain (score 4–6), and others (5 patients) had mild pain (score 1–3). Thus, overall, 14 out of 42 patients have mild and moderate chronic pain 3 months after surgery. The intensity of pain in mediastinitis patients significantly decreased at the three-months after chest reconstruction and it was lower than in patients with regular chest closure and no infection.

#### 3.3.1. Hazard ratio

Patients with chest wall reconstruction and conventional closure in all three groups had almost the same hazard ratio (0.75) for development severe pain in all time points post-surgery. However, regarding chronic mild-to-moderate pain in the third group with conventional closure and no chest inflammation, patients the probability increased three times more (0.25) 3-month point after cardiac surgery.

### 3.4. Inflammatory mediators and pain severity

Of the cytokines examined, all variables showed a significant relationship to the occurrence of sternal infection initially. It is consistent with severe inflammatory reactions due to mediastinitis. At baseline, we found a significant difference between the mediastinitis group (group 1) and groups with no infection (groups 2, and 3). The median values of serum cytokines: IL 1,6,17, TNFα, IFNγ, and RANTES were significantly raised (*p* < 0.0001) in deep sternal infection patients. While baseline levels of all cytokines were increased among patients who have developed a sternal infection, at discharge only the level of interleukin 6 remained significantly higher compared to no infection groups (92.6 ± 23.6 pg/ml vs. 71.6 ± 12.9 and 79.6 ± 17.7 pg/ml, *p* < 0.05). Regarding the second and third groups with no infection, the baseline and discharge values of cytokines were comparable without any significant differences (Table 3). C-reactive protein baseline level in group 1 also was significantly increased compared to groups 2 and 3 (176.8 ± 80.2 mg/L vs. 4.30 ± 1.67 mg/L and 4.11 ± 1.25 mg/L, *p* < 0.001). However, at the discharge time point, we did not find significant CRP differences between groups.

**Table 3:** Summary of cytokines expression at baseline and at discharge post-surgery.

Measure (pg/ml)	Mediastinitis LCWR group 1	No infection LCWR group 2	No infection CWC group 3	*p* Value
Baseline				
IL-1 β	103.7 ± 22.0	31.5 ± 12.1	32.6 ± 9.94	<0.0001****
TNF α	132.6 ± 39.4	60.4 ± 14.7	57.4 ± 13.8	<0.0001****
IFN γ	185.4 ± 58.7	90.0 ± 16.6	99.5 ± 24.6	<0.0001****
IL-6	108.5 ± 21.5	49.0 ± 13.9	52.1 ± 13.4	<0.0001****
IL-17	153.2 ± 52.2	60.5 ± 12.6	66.2 ± 16.4	<0.0001****
RANTES	8,600.7 ± 863.2	5,917.2 ± 606.0	6,022.2 ± 747.3	<0.0001****
Discharge				
IL-1 β	66.5 ± 25.5	49.8 ± 17.0	58.7 ± 19.7	0.12
TNF α	74.7 ± 20.0	66.1 ± 18.5	78.1 ± 20.9	0.26
IFN γ	145.9 ± 43.5	116.2 ± 31.7	129.7 ± 33.9	0.11
IL-6	92.6 ± 23.6	71.6 ± 12.9	79.6 ± 17.7	0.015*
IL-17	99.7 ± 30.7	79.0 ± 21.8	89.6 ± 16.9	0.085
RANTES	5,184.3 ± 2,326.1	6,180.4 ± 1,889.6	6,740.2 ± 1,757.6	0.12

Mean ± SD, *p*-values: unpaired *t*-test. IL, interleukin; TNF, tumor necrosis factor; IFN, interferon.

* *p*- value <0.01.

**** *p*- value <0.0001.

### 3.5. Sternal healing score and pain severity

**Figure 4:**
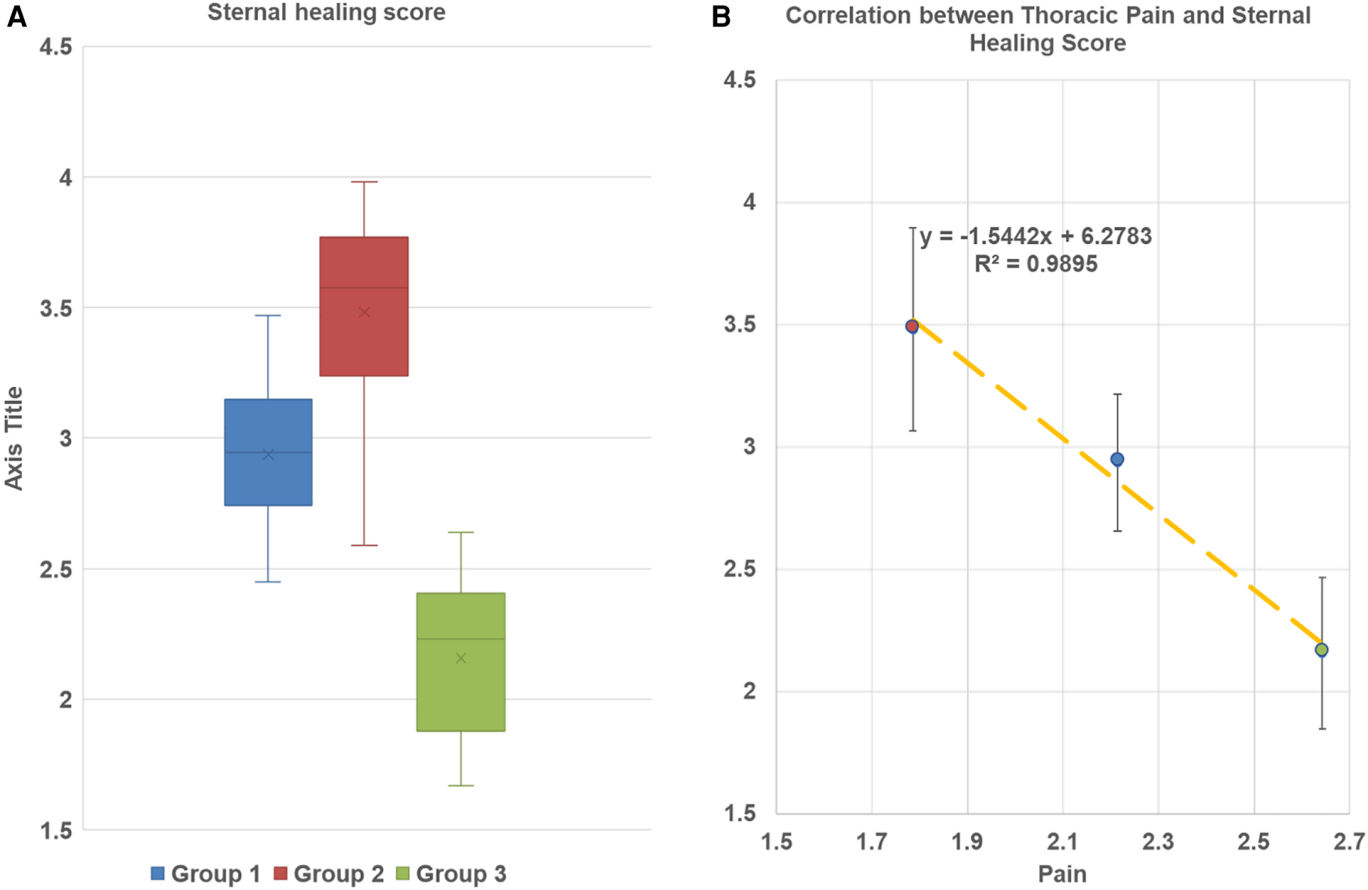
(**A**) Sternal bone healing score at 3-month follow up. Box and whisker plot diagram. The plots are demonstrated minimum and maximum values, first and thirs quartile median and mean of every group. No outliers werer found. Group 1 LCWR, longitudinal chest wall reconstruction with mediastinitis, Group 2 LCWR, longitudinal chest wall reconstruction with no infection), Group 3 CWC, conventional wire closure with no infection. (**B**) Correlation between thoracic pain and sternal bone healing score in all groups at 3-month follow-up.

Compared to CWC group 3, in LCWR, group 2 (no infection) we found better sternal healing scores at 3-month follow-up (3.48 ± 0.73 vs. 2.16 ± 0.62; *p* < 0.0001) and a higher percentage of patients with evidence of complete sternal union (42.8% vs. 21.4%; *p* < 0.001) (Table 4). In-group 1 with mediastinitis sternal healing score achieved 2.94 ± 0.52 and the percentage of patients with evidence of complete sternal union was 28.6%. These parameters were also higher than in the CWC group. Our regression analysis demonstrated a strong correlation between sternal bone healing score and pain intensity at different time points in all study groups (Figures 4A,B).

**Table 4:** Computer tomography scan assessment of sternal bone healing.

Groups	Mediastinitis LCWR group 1	No infection LCWR group 2	No infection CWC group 3	*p* Value
Sternal healing score at 3 months	2.94 ± 0.28	3.48 ± 0.41	2.16 ± 0.31	0.0001***
% patients with evidence of complete sternal union	(4) 28.6%	(6) 42.8%	(3) 21.4%	0.001**

Values presented as *N* (%), Mean ± SD, *p*-values: unpaired *t*-test.

** *p*- value <0.01.

*** *p*- value <0.001.

## 4. Discussion

In this study, we investigated chronic pain after cardiac surgery and its relationship with sternal infection and chest reconstruction technique. Our finding showed that 14 out of 42 patients still experienced chronic pain at 3 months post-surgery, with more patients experiencing pain in the group with no infection and conventional sternal closure than in the group with mediastinitis and chest reconstruction. Additionally, we found that cytokines were significantly related to the occurrence of sternal infection initially, with interleukin 6 levels remaining significantly higher in patients with sternal infection compared to those without infection. Also, we found a difference in ICU time between groups 2 and 3, with group 2 patients having a longer ICU stay. However, statistical analysis revealed that the difference is not significant. We believe that this lack of significance may be attributed to the fact that conventional sternal closure (group 3) is a widely accepted procedure that ICU doctors are very familiar with. In contrast, chest wall reconstruction is a relatively new technique, and doctors may have provided extra care to these patients. We did not observe any surgical or other complications between these groups.

Chronic pain after cardiac surgery is a significant issue characterized by allodynia and hyperalgesia, indicating its neuropathic origin. Post-surgery pain is subjective sensation, and every patient may experience it differently, depending on the type and severity of the procedure, anesthesia, physiological, and psychological factors (22, 23). Anxiety, emotional instability, and a pessimistic attitude can all affect pain perception. The precise development of CTP after cardiac surgery remains unclear. To minimize postoperative pain, many cardiac surgeons are modifying the traditional approaches, including chest closing techniques. While conventional wire closure is effective, it fails to provide stable compression and protect against chest instability, potentially increasing pressure at the lateral sternal edges (24). A sternal reconstruction technique with longitudinal rigid fixation provides even pressure distribution and reduces longitudinal motion, promoting better bone healing and stable sternal approximation to prevent post-cardiotomy pain. We believe that these biomechanical factors among others might contribute to decreased development of chronic pain and protect it from respiration, coughing, and ambulation. The CT scan data in all groups supported this hypothesis. While our study did not demonstrate complete sternal union in all rigid plate fixation cases, sternal union rates at 3 months were significantly higher after sternal reconstruction than with conventional wire closure.

Our study found that pain levels were higher on discharge day than later follow-up, and 14 out of 42 patients experienced persistent mild-to-moderate pain three months after cardiac surgery. It is consistent with previous research (3, 5, 10). Little is known about how mediastinitis treatment affects chest pain or how long it takes for chest pain to disappear after treatment. The process of chest wound healing is complex, involving a sequence of cellular and biochemical events that must be carefully orchestrated to ensure proper tissue repair. It is important to note that bacterial infections must be resolved before the wound can properly heal. Patients who are prescribed opioids for the management of chest pain may be at an increased risk for opportunistic infections and inadequate healing of sternal wounds, which are critical for recovery after cardiac surgery. Opioids are often used to manage chest pain, but we need better treatment options to decrease chest pain in cases of mediastinitis.

Cytokines play a critical role in initiating and maintaining inflammatory processes (25), and in addition to their effects on immune and inflammatory cells, they influence sensory neurons, which innervate thoracic tissue and mediate pain. During acute inflammatory phases, cytokines can induce sensitization via receptor-associated kinases and phosphorylation of ion channels, but the mechanism of cytokines action in recovery are unclear (26). Our study demonstrated that the impact of the increased levels of cytokines and CRP on postoperative pain presented only at the baseline time point in the group with mediastinitis. At post-treatment, the level of cytokines reduced except for IL 6 which remained significantly elevated at discharge time. IL 6 is produced in response to infections and its synthesis continues after the initial stages of inflammation, contributing to chronic inflammatory recovery (27). Thus, we expected that IL 6 levels would remain higher for a longer time. We did not find any significant correlations between pre- or intraoperative factors and a higher risk of pain. Some studies demonstrated relation between CTP and age and mass index (4, 28). But we did find a strong correlation between sternal bone healing and postoperative pain. Demographic values, duration and type of cardiac surgery procedures, length of ICU and hospital stay did not have a predictable impact on pain.

This study provides evidence that sternal reconstruction offers significant mechanical benefits, leading to better bone healing, reduced acute and chronic thoracic pain, and less need for opioids in the early postoperative period. Traditional surgical techniques allow for movement and separation of the sternal halves, which decreases the stability of sternal closure. The presence of sternal nonunion and movement may increase pain, which can impact recovery, respiratory function, mobility, and patient activity.

### 4.1. Limitations of the study

This study had a limited sample size for the group with mediastinitis due to the low incidence of deep sternal infection after cardiac surgery, and it was challenging to identify high-risk patients who matched the inclusion criteria. The confidence interval was not calculated to determine the expected prevalence when extrapolated to the entire population, and a multivariable logistic regression analysis was not conducted. Additionally, it would be beneficial to have a follow-up period of more than three months to gain a more comprehensive understanding of the outcomes.

## 5. Conclusion

The study's findings suggest the following: (a) among patients who underwent cardiac procedures with sternotomy, 14 out of 42 had chronic thoracic pain three months after surgery. However, in patients with previous sternal infection, only 5 out of 14 had pain, while 8 out of 14 of patients had pain after regular chest closure without infection, (b) the incidence and intensity of pain can be influenced by the surgical technique used for chest reconstruction, (c) treated mediastinitis does not appear to be a determining factor in the development of chronic chest pain, (d) except for interleukin 6, there is no correlation between cytokine levels and pain scores, even though interleukin 6 remains elevated for a long time after treatment, e) One of the significant strengths of the study is that it found a strong correlation between sternal bone healing and postoperative chest pain. The study's authors believe that further research that includes molecular and genomic factors is necessary to provide a more comprehensive evaluation of chronic chest pain after cardiac surgery.

## Data Availability

The original contributions presented in the study are included in the article/Supplementary Material, further inquiries can be directed to the corresponding authors.

## References

[1] Shemin EJ. The future of cardiovascular surgery. Circulation. (2016) 133:2712–5. 10.1161/CIRCULATIONAHA.116.023545 27324365

[2] Idataresearch.com. Available at: https://idataresearch.com/over-900000-cardiac-surgeries-performed-every-year-in-the-united-states/

[3] Meyerson J Thelin S Gordh T Karlsten R. The incidence of chronic post-sternotomy pain after cardiac surgery—a prospective study. Acta Anaesthesiol Scand. (2001) 45:940–4. 10.1034/j.1399-6576.2001.450804.x 11576043

[4] Kalso E Mennander S Tasmuth T Nilsson E. Chronic post-sternotomy pain. Acta Anaesthesiol Scand. (2001) 45:935–9. 10.1034/j.1399-6576.2001.450803.x 11576042

[5] Lahtinen P Kokki H Hynynen M. Pain after cardiac surgery: a prospective cohort study of 1-year incidence and intensity. Anesthesiology. (2006) 105:794–800. 10.1097/00000542-200610000-00026 17006079

[6] Marcassa C Faggiano P Greco C Ambrosetti M Temporelli PL. Italian association of cardiovascular prevention. Rehabilitation (GICR-IACPR). A retrospective multicenter study on long-term prevalence of chronic pain after cardiac surgery. J Cardiovasc Med. (2015) 16:768–74. 10.2459/JCM.0000000000000271 26258718

[7] Turk DC Wilson HD Cahana A. Treatment of chronic non-cancer pain. Lancet. (2001) 377(9784):2226–35. 10.1016/S0140-6736(11)60402-9 21704872

[8] Clark IC Allman RD Rogers AL Goda TS Smith K Chanas T Multimodal pain management protocol to decrease opioid use and to improve pain control after thoracic surgery. Ann Thorac Surg. (2022) 114:2008–14. 10.1016/j.athoracsur.2022.03.059 35430217

[9] Kracowski JC Hallman MJ Smeltz AM. Persistent pain after cardiac surgery: prevention and management. Semin Cardiothorac Vasc Anesth. (2021) 25:289–300. 10.1177/10892532211041320 34416847 PMC8669213

[10] Taillefer MC Carrier M Belisle S Levesque S Lanctôt H Boisvert A-M Prevalence, characteristics, and predictors of chronic nonanginal postoperative pain after a cardiac operation: a cross-sectional study. J Thorac Cardiovasc Surg. (2006) 131:1274–80. 10.1016/j.jtcvs.2006.02.001 16733157

[11] Gjeilo KH Klepstad P Wahba A Lydersen S Stenseth R. Chronic pain after cardiac surgery: a prospective study. Acta Anaesthesiol Scand. (2010) 54:70–8. 10.1111/j.1399-6576.2009.02097.x 19681771

[12] Clarke H Soneji N Ko DT Yun L Wijeysundera DN. Rates and risk factors for prolonged opioid use after major surgery: population based cohort study. Br Med J. (2014) 348:g1251. 10.1136/bmj.g1251 24519537 PMC3921439

[13] Raman J Lehmann S Sehr K De Guzman BJ Aklog L Garrett HE. Sternal closure with rigid plate fixation versus wire closure: a randomized controlled multicenter trial. Ann Thorac Surg. (2012) 94:1854–61. 10.1016/j.athoracsur.2012.07.085 23103010

[14] Madjarov JM Katz MG Fazal S Kumar A Madzharov S Handa A Use of longitudinal rigid sternal fixation in prevention and treatment of wound complications among high-risk patients after cardiac surgery. J Card Surg. (2021) 36:3155–62. 10.1111/jocs.15687 34056766

[15] De Feo M Renzulli A Ismeno G Gregorio R Della Corte A Utili R Variables predicting adverse outcome in patients with deep sternal wound infection. Ann Thorac Surg. (2001) 71:324–31. 10.1016/S0003-4975(00)02137-8 11216770

[16] Singh K Anderson E Harper GJ. Overview and management of sternal wound infection. Semin Plast Surg. (2011) 25:25–33. 10.1055/s-0031-1275168 22294940 PMC3140234

[17] Kleiman AM Sanders DT Nemergut EC Huffmyer JL. Chronic poststernotomy pain: incidence, risk factors, treatment, prevention, and the anesthesiologist’s role. Reg Anesth Pain Med. (2017) 42:698–708. 10.1097/AAP.0000000000000663 28937533

[18] Abboud CS Wey SB Baltar VT. Risk factors for mediastinitis after cardiac surgery. Ann Thorac Surg. (2004) 77:676–83. 10.1016/S0003-4975(03)01523-6 14759458

[19] Madjarov JM Katz MG Kane PN Madzharov S Robicsek F. Early surgical reconstruction of sternum with longitudinal rigid polymer plating after acute chest trauma. Ann Thorac Cardiovasc Surg. (2018) 24:324–7. 10.5761/atcs.cr.17-00156 29491197 PMC6300427

[20] Madjarov JM Katz MG Kumar A Gubara SM Madzharov S Madjarova S Median sternotomy after sternal reconstruction with bilateral longitudinal plating in a patient with osteoporosis. Heart Surg Forum. (2020) 23:E058–60. 10.1532/hsf.2783 32118544

[21] Stacy GS Ahmed O Richardson A Hatcherb BM MacMahona H Raman J Evaluation of sternal bone healing with computed tomography and a quantitative scoring algorithm. Open Med Imaging J. (2014) 8:29–35. 10.2174/1874347101408010029

[22] Doenst T Essa Y Jacoub K Moschovas A Gonzalez-Lopez D Kirov H Cardiac surgery 2016 reviewed. Clin Res Cardiol. (2017) 106:851–67. 10.1007/s00392-017-1113-2 28396989

[23] Morris BN. Survey of pain management in thoracoscopic surgery. J Cardiothorac Vasc Anesth. (2018) 32:1756–8. 10.1053/j.jvca.2018.02.013 29551282

[24] Lazar HL Salm TV Engelman R Orgill D Gordon S. Prevention and management of sternal wound infections. J Thorac Cardiovasc Surg. (2016) 152:962–72. 10.1016/j.jtcvs.2016.01.060 27555340

[25] Floege J Luscher B Muller-Newen G. Cytokines and inflammation. Eur J Cell Biol. (2012) 91(6–7):427. 10.1016/j.ejcb.2012.01.003 22365147

[26] Kany S Vollrath JT Relja B. Cytokines in inflammatory disease. Int J Mol Sci. (2019) 20(23):6008. 10.3390/ijms20236008 31795299 PMC6929211

[27] Tanaka T Narazaki M Kishimoto T. IL-6 in inflammation, immunity and disease. Cold Spring Harb Perspect Biol. (2014) 6:a016295. 10.1101/cshperspect.a016295 25190079 PMC4176007

[28] Bruce J Drury N Poobalan AS Jeffrey RR Smith WCS Chambers WA The prevalence of chronic chest and leg pain following cardiac surgery: a historical cohort study. Pain. (2003) 104(1–2):265–73. 10.1016/S0304-3959(03)00017-4 12855337

